# Surgery

**Published:** 1895-06

**Authors:** Charles A. Morton


### SURGERY.

There is, perhaps, no subject of more importance at the present time than the treatment of prostatic hypertrophy by castration. So many lives are made a burden by this disease in its more advanced form, and so much danger to life arises from its complications, that this method of treatment—almost free from risks and most encouraging in its results—is a great advance in surgery. As Dr. William White says, "the operation is almost painless, with a low mortality, and followed by no such unpleasant conditions as accompany persistent fistulous tracts, either suprapubic or perineal."

This method of treating enlarged prostate seems to have occurred to the minds of several surgeons about the same time. Dr. William White, of Pennsylvania, arranged for experiments to be made in connection with the subject at the end of 1892, and began to make suggestions early in the summer of the same year.[2] Mansell Moullin suggested it to a patient in November,

[1] *Brit. M. J.*, 1895, vol. i., p. 637.

1892.[1] The first operations were performed by Ramm in April, 1893,[2] independently of Dr. White's suggestion. Dr. White's ideas on the subject and the results of his experiments were not made public until they were embodied in a paper on Prostatic Hypertrophy, read before the American Surgical Association on June 1st, 1893, and published in the *Annals of Surgery*,[3] an abstract being published in the *British Medical Journal*,[4] the portion of it relating to prostatic hypertrophy being unabridged. Haynes, of Los Angeles, California, was the first to carry out White's suggestion in America.

The supposed analogy of uterine fibro-myomata to prostatic overgrowth, and the beneficial effect of oöphorectomy in the former, suggested the possibility to Dr. White that castration might cause atrophy of the enlarged prostate. The prostate is not really the homologue of the uterus, though the prostatic utricle is; and Dr. White recognises this, and says he does not argue for absolute homology between them. When Dr. White arranged to carry on experiments on this subject, he did so because of the supposed analogy between castration and the possible atrophy of the prostate it might produce, and oöphorectomy and its known effects on the diminution in size of uterine fibromyomata. His experiments consisted in the removal of the testes from dogs, with careful observations on the effect of the operation on the prostate. All the experiments are described in White's paper,[5] and I cannot do better than give the result in his own words. He says: "These results, which I believe may be relied upon, place beyond all peradventure the influence of castration (in the dog at least) upon the condition of the prostate, and show clearly that that operation is followed invariably, and with a promptness which I must confess was to me surprising, by atrophy first of the glandular and then of the muscular elements, and by a coincident reduction in both bulk and weight."

Before Dr. White undertook these experiments, evidence had been brought forward by Dr. Joseph Griffiths[6] to show that the size of the prostate varied with that of the testes, in order to prove that the prostate was essentially a sexual organ. Dr. White, at the time he commenced his experiments, did not know of these observations of Griffiths's—and, indeed, of Hunter's, long before; but they have a most important bearing on the question as to the use of castration in prostatic hypertrophy. John Hunter observed that in the mole the prostate gland in winter is "hardly discernible, but in the spring becomes very large and filled with mucus." With this Owen agrees, for he says: "The prostate gland in the mole begins to increase in February and acquires an enormous size, and conceals the urinary bladder towards the end of March;" and he describes a similar change in the prostate of the

[1] *Brit. M. J.*, 1894, vol. ii., p. 976. [2] *Centralbl. f. Chir.*, 1893, No. 35, p. 759.
[3] Vol. xviii., 1893, p. 152. [4] 1893, vol. ii, p. 575. [5] *Brit. M. J.*, 1893, vol. ii., p. 577.
[6] *J. Anat. & Physiol.*, vols. xxiii., xxiv., 1889–90.

hedgehog. Hunter was also the first to observe that the prostate of the castrated bull was atrophied. Griffiths, by careful naked-eye and microscopic examination of the prostate of the mole and hedgehog, confirmed the observations of Hunter, and shows from his own observations that the prostate in castrated cats, dogs, and other animals becomes greatly atrophied. Griffiths also refers to several instances in which double castration in men has led to marked atrophy of the prostate.

Even with all this evidence forthcoming, White at the end of his paper[1] says: "I have as yet no definite convictions;" and he did not seriously think of recommending the operation to patients, but waited for further information and the criticism of other medical men on his views—hence the fact, already referred to, that Haynes, of Los Angeles, and not White, was the first to carry out the treatment in America.

In connection with this question of the value of castration in prostatic hypertrophy, we must remember that the duration of the activity of the testes is very long. Men at quite an advanced age occasionally have children, and in the testes removed for hypertrophy of the prostate active spermatozoa have been found.[2]

M. Launois, of Paris, has lately studied the question from an embryological and physiological point of view, and finds[3] that in persons with only one testicle the corresponding side of the prostate is atrophied, and the same condition is present in cases of unilateral non-descent of the testicle; whereas in the absence of both testicles, or bilateral non-descent, the atrophy of the prostate is bilateral, as it is after bilateral castration or atrophy after orchitis.

A case occurred many years ago which, if reported, might have strengthened Dr. White's evidence in favour of castration for prostatic overgrowth very materially at a time when no clinical evidence was forthcoming. It was mentioned by Mr. Langton, at a meeting of the Clinical Society as lately as April 26th of this year. A gentleman, aged sixty-eight, who suffered from enlarged prostate had his right testicle removed for tuberculous disease, "with the result that the corresponding half of the prostate immediately diminished greatly in size." The left testis was removed three years later for the same disease, "and this operation was at once followed by diminution of the remaining half of the prostate. After the second operation, he could retain his water for hours, and enjoy a good night's sleep. He was still hale and strong."[4]

It is a very curious fact that some years ago Mr. Reginald Harrison[5] was asked by a patient to perform castration for hypertrophy of the prostate, but he would not consent, though, as a compromise, he divided the vasa deferentia. The patient stated that benefit immediately followed; but he was lost sight

1 *Brit. M. J.*, 1893, vol. ii., p. 578.

2 *Ibid*,, 1895, vol. i., p. 13, and Epitome, p. 42.

3 *Lancet*, 1894, vol. ii., p. 468, and *Ann. d. mal. d. org. génito-urin.*, Oct., 1894.

4 *Brit. M. J.*, 1895, vol. i., p. 979. 5 *Ibid.*, 1893, vol. ii., p. 709.

of, and what the further progress of the case was Mr. Harrison does not know. The method of treatment was suggested to Mr. Harrison by a case in which rapid atrophy of the testicle followed an operation for varicocele where the corresponding vas deferens had been accidentally divided in a young man. He considers that there is sufficient proof that division or rupture of the vas deferens is liable to be followed by atrophy of the corresponding testicle. Haynes of California, who performed the first castration for hypertrophied prostate in America, divided the vasa deferentia with "no definite results."[1] With regard to the effect of the division of the vasa deferentia on the growth of the prostate, Dr. Joseph Griffiths writes: "I have found that ligation or division of the vas deferens in dogs neither retards the growth of the testes in the young nor usually interferes with the maintenance of the spermatozoa-producing powers in the full grown."[2] This view is more fully elaborated by Dr. Griffiths in his recent lectures on the testes at the College of Surgeons.[3] He quotes Hunter's observations made in 1775 on a case in which the vas deferens was found obliterated, but the corresponding testicle, though its duct was occluded, was normal. Afterwards Curling, Gosselin, and Godard concluded, from experiments on dogs and *post-mortem* observations in men, that atrophy of the testicle did not follow obliteration of the vas deferens. Griffiths in his experiments shows that not only does occlusion of the vas deferens fail to produce atrophy of the fully-developed testis in dogs, but that it does not interfere with its development in puppies. But the results were not quite uniform in Griffiths's experiments, for in one case atrophy was produced. Much seems to depend on the activity of the gland after occlusion of the vas deferens. Brissaud[4] has shown that in rabbits, after ligation of the vas deferens, different results follow according to the conditions under which the animals are placed after the operation. If placed by themselves, nothing more than a passing enlargement of the epididymis and testicles occurred; but if allowed access to the does, "after a period of super-activity" the testicles become atrophic. Now, in the case of obliteration of the bile-duct we get atrophy of the liver-cells and cirrhosis from backward pressure in the duct. Why, then, does this not always occur in the testes after ligation of the vas deferens? Griffiths suggests that it is because of the great length of its tubules and the slow secretion of spermatozoa, or from the absorption of seminal fluid taking place in proportion to its rate of secretion. At the end of his lectures Dr. Griffiths says: "With regard to the suggestion made by Mr. Reginald Harrison of division of the vas deferens in cases of enlarged prostate I have not sufficient experience to arrive at a conclusion; but, seeing that obliteration of the vas deferens has so

[1] Referred to in White's paper, *Brit. M. J.*, 1894, vol. i., p. 1353.
[2] *Brit. M. J.*, 1893, vol. ii., p. 765. [3] *Lancet*, 1895, vol. i., p. 917.
[4] *Arch. de physiol. norm. et path.*, 1880, p. 769.

little effect upon the structure and secretion of the testis, it must be doubtful whether the operation will suffice to influence the enlargement of the prostate."

Dr. White is at present engaged in a series of experiments on dogs in order to find out the effect of ligation of the arteries of the cord or the entire cord on the size of the prostate, and expects to report them in full in a paper on the subject which he is preparing at the invitation of the American Surgical Association.[1] But, as Dr. White points out, all such methods really act, if at all, by first causing atrophy of the testicles. The sentimental objection to castration has been overcome in two instances by the insertion of celluloid balls in place of the removed testes. Mr. William H. Bennett records[2] some observations which show that, in some cases at any rate, atrophy of the testicle does not follow aseptic ligation of all the structures of the cord—vas deferens, spermatic vein, and spermatic artery.

Quite recently the question has been raised whether unilateral castration might not be effective in relieving cases of prostatic overgrowth. Ramm suggests that in cases where an asymmetrical hypertrophy can be distinctly diagnosed it would be more conservative to excise only the corresponding testicle at first.[3] In the *British Medical Journal*[4] of March 2nd, 1895, is a letter from Dr. White asking for information on the subject. He said that the existing evidence is scanty, but seems to point clearly to one-sided atrophy of the prostate as a common result of removal of one testicle. Some experiments which he is now making on dogs corroborate this view, as does the observed condition of the prostate in monorchids. If it is found that the diminution in size extends to the other lobe, or if the shrinking of one lobe will lessen the obstruction, it is obvious the operation might be extended to a much larger number of patients at an earlier stage of this disease. Mr. Hurry Fenwick at once replied[5] by a record of twenty cases in which he had examined the prostate after unilateral castration, or where the testicle on one side was absent or atrophied, which do not support the view that unilateral atrophy of the prostate follows one-sided castration. A case reported by Mr. Baldwin[6] shows, from *post-mortem* evidence, that the prostate was of almost equal size on the two sides, though one testicle was very minute. I have myself observed very marked atrophy of one side of the prostate after unilateral castration.[7] Remondino, in California, has seen recovery from a gradually increasing prostate enlargement (which had been recognised for three years) after unilateral castration.[8]

I now turn to the clinical aspect of the question to see

[1] *Brit. M. J.*, 1895, vol. i., p. 448. [2] *Lancet*, 1895, vol. i., p. 1018.
[3] *Brit. M. J.*, 1895, vol. i., Epitome, p. 41.
[4] *Brit. M. J.*, 1895, vol. i., p. 508. [5] *Ibid.*, p. 529. [6] *Ibid.*, p. 698.
[7] *Ibid.*, p. 1035. [8] *Ibid.*, p. 786.

whether the hope raised by experimental evidence is made a certainty by the actual trial of the method. Dr. White, writing at the end of last February,[1] says: "I have knowledge now of thirty-eight cases of this operation. I am in communication with the operators in eighteen of the unpublished cases. I have operated four times myself. I may state that, so far as I have been able to learn, all the cases operated on in this country have been benefited, no matter what the form or character of the prostatic growth; none of them have relapsed or returned to the clinical conditions which existed previous to the castration; in none of them have any changes in intellectuality been observed." Such a statement as this coming from Dr. White—a man so cautious that he abstained from putting into practice a method of treatment fairly well supported by experimental and collateral evidence until further information was forthcoming, or other medical men had had an opportunity of criticising the method—seems to me to be sufficient to establish castration for prostatic hypertrophy on clinical evidence.

Mr. Mansell Moullin has operated upon six cases,[2] and has records of seven others in which he has been consulted or which have been communicated to him by friends, making a total of thirteen. With the exception of two all are doing well, and are not suffering in any way from mental troubles. Of the two exceptions, one died from cardiac failure on the eleventh day, and one, at the age of eighty-two, nine months after an operation which was most successful. Mr. Moullin points out that the success of the operation will depend on the condition of the bladder and prostate. If the former is ruined by long-continued septic cystitis and catheterism, it cannot be restored. If the enlargement of the prostate is in the adenomatous stage, recovery is rapid; but if fibroid—very firm and hard—it may be some months before there is any definite improvement. Dr. Joseph Griffiths[3] has examined the enlarged prostate microscopically after its diminution in size on the eighteenth day after castration, and finds marked atrophy of the glandular tissue—a change similar to that which takes place in the dog after castration. He also draws attention to the fact pointed out by Mansell Moullin that it is in the earlier stage of prostatic overgrowth, when the enlarged prostate is mainly adenomatous, that castration is most likely to do good, and not in the later stage, when degenerative changes set in which ultimately convert the new tissue into a mass of more or less dense fibrous tissue containing only the atrophied remains of the glandular and muscular elements. Seven cases are reported by Mr. Hurry Fenwick,[4] and he says neither he nor his patients have been disappointed with the results.[5] He calls attention to the fact that when the bladder has been emptied for a long time by catheter only and is in a condition of

[1] *Brit. M. J.*, 1895, vol. i., p. 448. [2] *Ibid.*, p. 1,064. [3] *Ibid.*, p. 579. [4] *Ibid.*, p. 578. [5] *Ibid.*, p. 1,065.

advanced atony, we cannot expect it to regain power after castration. Dr. Faulds[1] records several cases in which mania or mental weakness followed the operation within a week; but, looking at the records of other surgeons, I cannot help thinking Mr. Moullin is right when he says that Dr. Faulds[2] must have met with some strange succession of coincidences, and that the mental effect must have been due to the disturbance of the anæsthetic and operation in an old person, and not to the removal of the testes in particular, as in two of the cases only one testicle was removed.

Space will not allow me to give an abstract of anything like all the cases published, and I must be content with giving in tabular form as many as I can find and a fuller abstract of a few of special interest. The first cases operated on were those of Ramm, of Christiania.[3] One patient, aged seventy-three, had suffered from difficulty in micturition for fifteen years and once from retention. During the year before the operation he passed urine almost hourly. The prostate was the size of a medium-sized orange. After bilateral castration on April 3rd, 1893, the gland at once began to atrophy, and the report a year later says: "Now he passes water as well as he ever used to, and has only to empty his bladder twice during the night," and the prostate "is a small flat mass." The effect of the operation in the other case is even more striking, as the condition before operation was so much worse. The patient was sixty-seven years of age, and had suffered from difficulty in micturition for fourteen years. He had twice had retention; and the last time—a year before castration—the bladder had been punctured above the pubes, and cystitis had been present from that time. The prostate was so large that its upper border could only just be reached by the finger. Bilateral castration was performed on April 25th, 1893, and six weeks after he could pass a good stream of urine. A year later he passed water only four or five times during the day and once at night, and all difficulty and the cystitis had completely disappeared. The prostate had greatly atrophied.

The first operation recorded in this country is that performed by Mansell Moullin on June 21st, 1894. The patient was eighty-one years of age, and was admitted to the London Hospital suffering from retention, which had occurred several times before. The prostate was enormously enlarged, and no catheter could be passed. Suprapubic aspiration was performed, and after that the power of micturition very imperfectly returned, and cystitis set in. The day after castration the urine came more freely; ten days later the prostate was smaller; and three weeks after the operation it had disappeared, and an ordinary catheter passed with ease.

[1] *Brit. M. J.*, 1895, vol. i., p. 974. [2] *Ibid.*, p. 1,064.

[3] *Centralbl. f. Chir.*, 1894, No. 17, p. 387, and 1893, No. 35, p. 759; and *Brit. M. J.*, 1894, vol. i., p. 1052.

| | Name of Operator. | Date of Operation. | Where Published. | Age. | History of Case. |
|---|---|---|---|---|---|
| 1 | Dr. Francis L. Haynes, of Los Angeles, California | 1893 | *Buffalo M. & S. J.*, vol. xxxiii., 1894, p. 481 | — | Symptoms of two years' duration. |
| 2 | Do. | 1893 | Do. | — | — |
| 3 | Dr. William White | Jan. 31st, 1894 | *Brit. M. J.*, 1894, vol. i., p. 1353 | 69 | Passed no urine except by catheter for years. Occasional hæmaturia. |
| 4 | Dr. Fremont-Smith | Jan. 17th, 1894 | *Ann. Surg.*, vol. xx., 1894, p. 52 | 69 | Mild symptoms for a year. For two months "irregular fevers," frequent and painful micturition. Frequent attacks of retention. |
| 5 | Mr. Hurry Fenwick | 1894 | *Brit. M. J.*, 1895, vol. i., p. 578 | 76 | Some years of feeble prostatic micturition. |
| 6 | Do. | 1894 | Do. | 70 | Symptoms six years. Attacks of retention. |
| 7 | Do. | 1894 | Do. | 76 | Repeated attacks of retention. |
| 8 | Do. | 1894 | Do. | 81 | Emptied bladder by catheter for nine years: six or seven times at night and three during the day. |
| 9 | Do. | 1894 | Do. | 60 | Three years' dysuria, with occasional use of catheter. Prostatic abscess formed and burst into urethra. Then retention, followed by catheterisation for a week. |
| 10 | Dr. James Swain | Sept. 11th, 1894 | *Brit. M. J.*, 1895, vol. i., p. 12 | 73 | Dysuria for five years, and catheterisation needful greater part of this time. Twice retention. |
| 11 | Ramm (additional case) | — | *Brit. M. J.*, 1895, vol. i., Epitome, p. 41 | 57 | Dysuria ten years; retention ten days, relieved by catheterisation. |
| 12 | Koren | — | *Brit. M. J.*, 1895, vol i., Epitome, p. 42 | 66 | Symptoms for a year. |
| 13 | Mr. Mansell Moullin (additional cases at Clinical Society) | — | *Brit. M. J.*, 1895, vol. i., p. 979 | 74 | — |

| | Condition of Patient at Time of Operation. | Bilateral or Unilateral Castration. | Result. |
|---|---|---|---|
| 1 | — | B | "Practically cured." |
| 2 | "Desperate case requiring catheterisation every two hours, complicated by intense cystitis and by morphinism acquired as result of frightful suffering." | B | "Cystitis has disappeared, and one-third of urine passed spontaneously. Catheter used about four or five hours; morphinism cured." |
| 3 | Prostate size of orange. Urine loaded with mucus and offensive. Catheter passed 9½ inches before urine flows, and passage difficult and very painful. | B | Fourteen weeks after operation had not urinated spontaneously, but prostate reduced to almost normal dimensions. Urine flows when catheter passed for 8 inches, and is easy and painless. Urine normal. |
| 4 | Urine purulent and offensive. Alarming weakness. Six ounces of residual urine. Mental condition weak and melancholic. Nocturnal micturition twelve or fourteen times. | B | Six weeks after operation: No retention. Only a few drachms of residual urine; hardly any pus in urine. Nocturnal micturition four to six times. Gained strength and weight, and mental condition better. Two months later said to have no trouble or pain with micturition. |
| 5 | Retention. Prostate unusually hard and large. | B | Prostate diminished; stream improved; and micturated less often. |
| 6 | Prostate not enlarged *per rectum*, but lateral lobes seen by cystoscope to be much upraised towards the bladder. Cystitis. | B | "Stream markedly improved in force, and no symptoms except slight variability in the power of the bladder remained. Urine cleared in first week." |
| 7 | "Catheterisation most difficult. Prostate only moderately enlarged towards the rectum. Supra-pubic drainage for months, without improvement to the cystitis." | B | "Cystitis rapidly diminished, and voluntary micturition recovered in a fortnight," with good stream. Prostate slightly smaller in three weeks. |
| 8 | Large elastic prostate. Increasing difficulty in passing catheter. Cystitis present. | B | Report three weeks after operation gives diminution in size of prostate, and marked diminution in frequency with which catheter had to be passed. Cystitis also much better. |
| 9 | Prostate large laterally and elastic. | B | Recovery of voluntary micturition on third day. Slight shrinking of prostate in a month. Pain disappeared. Frequency of micturition became normal. |
| 10 | Retention. Bladder aspirated. Then in a a few days again possible to pass catheter. Urine offensive. Prostate as large as an orange; urine did not flow through catheter until it had been passed for 9½ inches. | B | Prostate diminished to size of "large horse-chestnut." All difficulty in passing urine disappeared; nocturnal micturition ceased; urine became normal. |
| 11 | Symmetrically enlarged prostate; dilated bladder; severe cystitis. | B | Prostate atrophied and all difficulty in micturition ceased. |
| 12 | Prostate much enlarged. | B | Prostate atrophied and patient relieved of all symptoms. |
| 13 | Enormous prostate; urine ammoniacal; intense strangury; persistent cystitis. Micturition fourteen times at night in spite of the frequent use of morphine. Bladder only held two ounces. | B | Six weeks after operation: Urine only contained a faint cloud of mucus. Strangury almost gone; bladder held 4 oz. Slept 1½ hours, and had given up morphine. Prostate smaller. General condition of patient immensely improved. |

| | Name of Operator. | Date of Operation. | Where Published. | Age. | History of Case. |
|---|---|---|---|---|---|
| 14 | Mr. Mansell Moullin (additional cases at Clinical Society) | — | *Brit. M. J.*, r895, vol. i., p. 979 | — | Repeated attacks of cystitis and hæmaturia. No urine passed except by catheter for fifteen months, and catheter passed every hour day and night. |
| 15 | Dr. Piercy | Aug. 8th, 1894 | *Med. Rec.*, vol. xlvii., 1895, p. 239 | 71 | More frequently occurring attacks of retention. |
| 16 | Dr. Walker | Jan. 23rd, 1895 | *N. York M. J.*, vol. lxi., 1895, p. 481 | 65 | Symptoms for eight years; used a catheter for a year. |
| 17 | Do. | Feb. 6th, 1895 | Do. | 71 | Increasing difficulty in micturition for fourteen years. Uses catheter, often more frequently than every hour. |
| 18 | Do. | Feb. 26th, 1895 | Do. | 63 | Symptoms seven years. Four years before castration median prostatic growth removed with only temporary benefit. |
| 19 | Dr. Faulds | Dec. 24th, 1894 | *Brit. M. J.*, 1895, vol. i., p. 974 | 66 | Catheter life for three years. |
| 20 | Do. | Jan. 3rd 1895 | Do. | 71 | — |
| 21 | Do. | — | Do. | — | Six years' dysuria, with blood and pus in urine. |
| 22 | Do. | — | Do. | — | Symptoms for four years. |

### OBSTETRICS.

| | Condition of Patient at Time of Operation. | Bilateral or Unilateral Castration. | Result. |
|---|---|---|---|
| 14 | Very large prostate. | B | On the 10th and 11th days after castration, blood-stained urine passed spontaneously. On the 11th day the patient died with extreme dyspnœa. At the P.M. the heart was found fatty and the kidneys cirrhotic. The bladder contained a calculus. The prostate had begun to atrophy, as shown by the wrinkled mucous membrane lying over it. |
| 15 | Prostate immensely enlarged. | B | In two months prostate diminished one-half; no trouble with micturition. |
| 16 | Aggravated cystitis. Night much disturbed by micturition. | B | Prostate atrophied; urine became nearly clear; all difficulty in micturition disappeared. |
| 17 | Very much enlarged prostate and "usual cystic symptoms." | B | Cystitis nearly disappeared; holds water much longer, but has to use catheter occasionally. Too early for complete report. |
| 18 | "Almost constant reminder that he has to void urine." | — | March 29th patient writes: "But little pus in urine; entire subsidence of pain; urinates but about once in twenty-four hours, and does not have to use catheter." |
| 19 | Twelve ounces residual urine, mixed with pus and blood. Very large prostate. | B | A week after, dysuria less, but died of hemiplegia Jan. 19th, 1895. |
| 20 | — | B | Died on sixth day of acute mania. |
| 21 | Enlarged prostate. | B | Mania came on, and died on twelfth day. |
| 22 | — | B | Report thirty days later gives no improvement. |

CHARLES A. MORTON.

[2] *N. York M. J.*, vol. lxi., 1895, p. 71.

